# Personal and Social Responsibility Development in a Volleyball Hybrid Positive Youth Development Program: A Mixed Methods Approach

**DOI:** 10.3389/fpsyg.2021.675532

**Published:** 2021-08-11

**Authors:** Antonio Muñoz-Llerena, Elena Hernández-Hernández, Antonio García-de-Alcaraz, Pablo Caballero-Blanco

**Affiliations:** ^1^Physical Education and Sport Department, Universidad de Sevilla, Seville, Spain; ^2^Sport and Computer Science Department, Universidad Pablo de Olavide, Seville, Spain; ^3^SEJ-570 MOTIVA2 Research Group, Universidad Pablo de Olavide, Seville, Spain; ^4^Faculty of Educational Sciences, Universidad de Almería, Almería, Spain; ^5^LFE Research Group, Universidad Politécnica de Madrid, Madrid, Spain

**Keywords:** pedagogical model, teaching personal and social responsibility model, sport education model, mini-volleyball, competitive sport

## Abstract

Positive Youth Development (PYD) programs are being used to foster holistic growth in children and adolescents. The hybridized application of two or more programs of this type has acquired special relevance in recent years. Although their application is common in the school context, there are few research studies that attempt their implementation in an extracurricular context. This study analyzed the effects of an intervention based on a hybrid PYD program on personal responsibility (PR) and social responsibility (SR) in youth volleyball players in an extracurricular context. A hybrid program was applied during the competitive season, with a total of 37 sessions with 30 girl students (15 experimental and 15 control) aged between 8 and 10 years (*M* = 8.87, *SD* = 0.82). A convergent mixed methods design was applied to integrate the following: (a) semi-structured interviews and field notes and (b) personal and social responsibility questionnaires. The results indicated that the implementation of the hybrid program appeared to yield a positive perception of learning in both the participants and the coach. Although there were no statistically significant intergroup or intragroup differences, the findings suggest that the hybrid program seems to be effective in fostering PR and SR in youth girl volleyball players.

## Introduction

The use of Positive Youth Development (PYD) programs is currently on the rise. PYD, which has its origins in positive psychology (Caballero, 2015), is a way of understanding development rather than a specific construct and is used as an umbrella term to refer to the ways in which youth accumulate optimal developmental experiences in organized activities (Holt and Neely, 2011). Among these organized activities, sports are the most widespread among young people (Larson and Verma, 1999) and are a privileged context for promoting PYD (Fraser-Thomas et al., 2005; Petitpas et al., 2005; Escartí et al., 2009).

There are many potential benefits of sport-based PYD (SBPYD) programs. On a physical level, there may be improvements in the cardiovascular system, muscular strength and resistance, flexibility, bone structure and weight control, and a lower risk of suffering from cardiovascular diseases, diabetes, obesity, osteoporosis, stroke, depression, or cancer. On a psychological/emotional level, opportunities are offered to experience challenge, fun, and enjoyment. SBPYD programs can also increase self-esteem, satisfaction, and happiness and can also decrease stress. On a social level, such programs promote citizenship, social success, positive interrelationships, and the development of skills such as leadership, cooperation, responsibility, empathy, or self-control. On an intellectual level, there is a positive relationship between these programs and academic performance, class attendance, study time, and university attendance (Fraser-Thomas et al., 2005; Petitpas et al., 2005; Theokas et al., 2008; Weiss et al., 2013, 2020; Ivy et al., 2018). However, SBPYD programs may not always foster all of these benefits.

There are different factors that must be taken into account for the successful implementation of SBPYD programs. PYD study states that the elements needed to be present in a sports program to achieve PYD are as follows: the climate of the program and the contents to be taught (Escartí et al., 2009); a mastery-oriented and caring climate, peer/adult supportive relationships, and opportunities to learn life skills (Weiss and Wiese-Bjornstal, 2009); or the individual learner (internal and external assets and autobiographical experiences), the learning contexts (school, sport, family, vocational, and extracurricular), and the transfer contexts (factors, e.g., similarity of context, opportunities to use skills, support and rewards for transfer, and psychological processes influencing transfer, e.g., unconscious personal reconstructions, confidence, or level of engagement) (Pierce et al., 2017). Indeed, Petitpas et al. (2008) state that, to develop PYD, sports programs must present voluntary and motivating activities that require effort and commitment and have clear rules, which are used as a metaphor to teach life skills and their application in the lives of youths, all of which should occur within a caring and safe environment led by a respectful adult. On this matter, Holt et al. (2017) established a model of PYD through sport, stating that a program should include a PYD climate (based on relationships between athletes and peers, parents, and other adults) and a focus on life skills program (employing life skill-building and transfer activities) in order to achieve personal, social, and physical PYD outcomes. According to their model, enhancing these outcomes “will facilitate transfer and enable youth to thrive and contribute to their communities” (Holt et al., 2017, p. 38).

Within competitive youth sport, Santos and Martinek (2018) established four strategies for coaches to promote its educational potential: to assume the double objective of improving sports skills while learning life lessons and building a positive character, to convert PYD into specific, adapted behaviors, to integrate a PYD-based approach into training sessions, and to maintain a balance between winning expectations and the PYD intervention regardless of the seasonal demands.

At present, there are different intellectual currents derived from the PYD theoretical frameworks, related to how PYD can contribute to better sports experiences for youth that connect to their daily lives in current society. Some, like critical PYD (Gonzalez et al., 2020), are focused on adopting a critical perspective on PYD, recognizing the values of youth and their capacity to challenge inequities, and transforming society and its structures to erase oppression. Others reflect on the long-term implications of SBPYD programs and present solutions to support and promote positive individual behavioral changes in the long term, such as the MINDSPACE (Messenger, Incentives, Norms, Defaults, Salience, Priming, Affect, Commitment, and Ego) model (Whitley, 2021). One way to apply SBPYD programs is through pedagogical models. Two of the most utilized ones from the scientific studies that align with the guiding principles of PYD are the teaching personal and social responsibility model (TPSR; Hellison, 2011) and the sport education model (SE; Siedentop, 1998; Siedentop et al., 2004). These pedagogical models have been applied with positive results in different Spanish contexts and with different populations, ages, sports, and educational levels. They have become the pedagogical models that are most commonly used by researchers in Spain (Escartí et al., 2010a,b; Meroño et al., 2015, 2016; Fernández-Gavira et al., 2018; Camerino et al., 2019; Manzano-Sánchez and Valero-Valenzuela, 2019; Manzano-Sánchez et al., 2019, 2020; Valero-Valenzuela et al., 2020a; Carreres-Ponsoda et al., 2021).

The TPSR model is one of the most relevant models aligned with the PYD framework, owing to its implementation in different countries, the wide variety of research studies about it, and the ease of its hybridization with other models (Escartí et al., 2009; Caballero Blanco et al., 2013; Pozo et al., 2018; Baptista et al., 2020; Sánchez-Alcaraz et al., 2020). It is characterized by strong instructor-participant relationships through specific guidelines, along with empowerment and personal and group reflections, carried out gradually. These tools aim for those involved to take responsibility for their actions at a personal and social level (Hellison et al., 2008). A characteristic framework was, therefore, designed that included the core values of the model, premises, levels of responsibility, instructor responsibilities, application format, suggested strategies, problem-solving, and evaluation (Hellison, 2011).

The SE model was designed to provide authentic and enriching sports experiences (Siedentop, 1998). The basic characteristics of this model arise from the particularities of sports (Siedentop et al., 2004), which make it possible to pursue objectives beyond those of learning a given technical skill. For the creators of this model, a competent athlete will possess the necessary skills to participate satisfactorily in these and other activities that arise, understanding them, and are being able to apply and execute appropriate strategies depending on the complexity of the situation.

There is no pedagogical model that is effective in all contexts and contents (Lund and Tannehill, 2010). In the words of Martinek and Hellison (2016, p. 13), “One size does not fit all (…). Drawing on personal strengths and available resources and augmenting interpersonal processes between youth and staff will be essential for (…) successful implementation.” To overcome these limitations, different pedagogical models have begun to be combined when implemented to adapt them to the intervention context, enhancing their educational effects, and reducing the limitations that may exist in the application of any given model in isolation (Haerens et al., 2011; González-Víllora et al., 2019). According to González-Víllora et al. (2019, p. 13) “hybridizations could be considered a new and innovative trend that is necessary to increase the benefits and possibilities for the implementation of pedagogical models.” A previous research study has shown that these benefits have a positive impact in two main areas—sports skills and psychosocial variables (psychological, social, and personal development)—as long as a logical and appropriate use of the intervention is carried out (Fernández-Río et al., 2016). The proposal presented in this study analyzes personal responsibility (PR) and social responsibility (SR), a variable that falls within the psychosocial field proposed by the previous authors.

Hybridizations of TPSR or SE with different pedagogical models are especially frequent. The separate combination of TPSR—or SE—with other models has obtained positive results when applied in educational contexts in sports (Hastie and Curtner-Smith, 2006; Pritchard and Mc-Collum, 2009; Mesquita et al., 2012; Caballero-Blanco, 2015; Araújo et al., 2016, 2019; Valero-Valenzuela et al., 2020b), which strengthens support for the usefulness of designing hybrid programs that combine the benefits of both models (Menéndez-Santurio and Fernández-Río, 2016a,b, 2017; Fernández-Río and Menéndez-Santurio, 2017; Muñoz-Llerena et al., 2019). In the previous research studies, the feasibility of hybridizing TPSR and SE has been analyzed, with different studies considering SE suitable to combine with TPSR as long as the implemented programs are designed to offer the opportunity to experience all five levels of responsibility, noting that “the pressure and perceived importance of competitive sports can be useful in testing the depth of commitment to, and engagement with, the learning outcomes of TPSR” (Gordon, 2009, p. 15). Such studies have also pointed out that these two models share a common theory of learning (the constructivist theory; Hastie and Buchanan, 2000).

However, Gordon (2009) considered that tensions might arise in the implementation of both models together in physical education (PE) classes when it comes to the use of games within sports practice and by the emphasis placed on internal (TPSR) vs. external (SE) sources of authority, as they have different purposes: SE aims to promote good sports skills, while TPSR aims to help youngsters become better people. In PE, a decision must, therefore, be made about which model should be prioritized, rather than attempting to meet the goals of both.

Outside the regulated context of PE classes, the application of hybrid programs has seldom been addressed in scientific studies (González-Víllora et al., 2019). Only Muñoz-Llerena et al. (2019) have proposed a design for a PYD program hybridizing TPSR and SE for application, especially in team sports in extracurricular competitive contexts: the Team Sports Positive Development Program (TESPODEP). This hybridization was carried out bearing in mind the greater possibilities offered by hybridized pedagogical models to adapt to the intervention context and increase their effects on the participants. Using TPSR as a way to promote psychosocial development, complemented with the specific strategies of SE, contributes to promoting sports development while reinforcing the psychosocial gains obtained. In addition, the hybridization of these models allows the intervention to be adapted to a competitive team sports structure, which makes it possible to achieve, with the same intervention, improvements in a large number of participants and in a context in which winning is usually more important than the holistic development of the players.

To verify the benefits of an implementation based on this program, this study analyzed the effects of this hybrid TESPODEP program on PR and SR in youth volleyball players in an extracurricular context. The rationale for this research study was to provide evidence for how the implementation of a hybrid TPSR+SE program might affect players in an out-of-school competitive team sports club, helping to fill the existing gap in the study while offering a flexible model specifically designed to be applied in team sports.

Volleyball was chosen because of its differentiating characteristics from other sports. Unlike most team sports, volleyball is conditioned by the impossibility of retaining the ball; thus, it can only be played by hitting. This limitation influences the importance of technique in ball control and, therefore, the dependence between the collective contacts to win the point. The collaboration between teammates is crucial for scoring, when compared to other sports where a single player could score. In volleyball, except for the serve, the rest of the actions are sequential and depend on the previous and following ones, so cooperation or teamwork is of special relevance. Furthermore, in the initial stages the technical quality is lower, so it becomes essential to emphasize cooperative tactical behaviors, which compensate the limited mechanical efficiency, to achieve continuity in the game. Finally, the fact of not being able to hold the ball increases the timing demands of the game, which reinforces the need to achieve an adequate emotional state that encourages cooperation, decision-making, and autonomy in favor of the aims of the team.

Competitive youth sport “can serve as an appropriate context conducive to PYD” (Ferreira dos Santos et al., 2018, p. 229), being a fertile platform that can lead to developments in life skills such as perseverance, respect, teamwork, or leadership (Santos and Martinek, 2018). It was chosen based on the need to increase the research field with more studies that employ rigorous methodology and the suitability of competition as a perfect setting to implement SBPYD models, due to the reduced number of participants and its voluntary access (Carreres-Ponsoda et al., 2021) and the focus on empowering personal and social strengths (Harwood and Johnston, 2016; Jørgensen et al., 2020). In addition, there is a need for more research studies to clarify our understanding of the development of life skills through competitive SBPYD programs (Jacobs and Wright, 2018).

The study was put into practice based on a series of research questions and hypotheses. The main hypothesis was that the proposed intervention would promote the development of PR and SR in the participants. The research questions considered were as follows: What are coaches' and athletes' perceptions concerning teaching and learning personal and social responsibility as a consequence of the intervention? What is the impact of the TESPODEP program on athletes' personal and social responsibility outcomes? To what extent do the quantitative responsibility data obtained agree with the findings of the interviews and field notes on the development of personal and social responsibility perception in the players?

## Materials and Methods

### Context

The main goal of the intervention program was to train participants to become enthusiastic, competent, literate, and responsible sportspersons and be capable of leading a team and making decisions that benefit the whole group. The intervention took place in two subsidized schools in Seville (Spain), which presented similar sociodemographic profiles and belonged to neighborhoods with an upper-middle socioeconomic level.

In Seville, federated sports clubs usually have a defined framework or guidelines for training programs. However, in clubs that are not part of a federated competition, it is common for each coach to work in his or her own way, and there is no consensus or guide on how to coach a team. In general, coaches tend to be focused on achieving victory rather than on the process of the personal development of each athlete. In the Catholic Schools Competition, where the intervention took place, they are somewhat more flexible and let the coach be the one who sets the model to follow. In this way, everything depends on the level of training and knowledge of the coach of this type of methodologies, far from those centered on execution models, typical of the courses of coaches.

### Research Design

**Figure 1:**
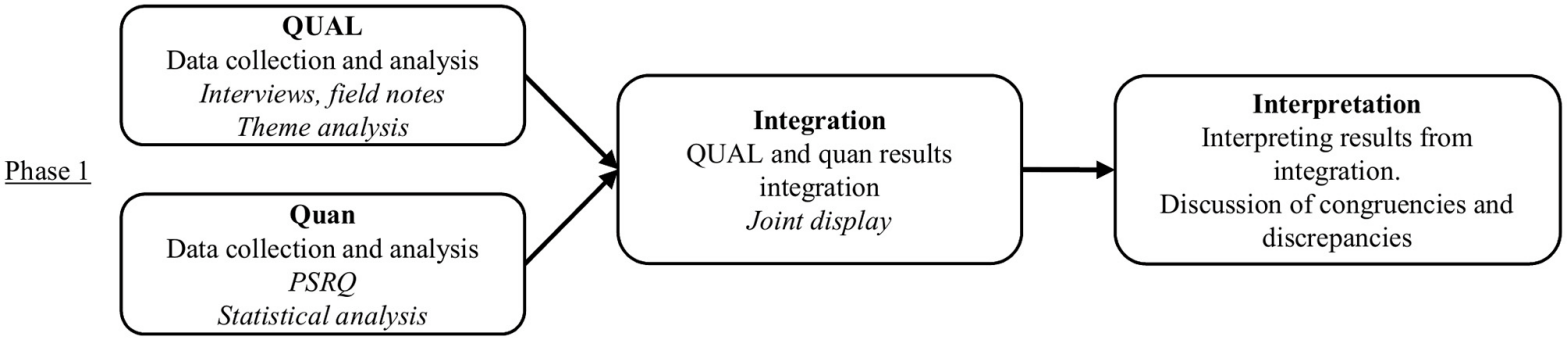
Convergent mixed methods design process.

This study used a convergent mixed methods design (Figure 1), because this type of design acknowledges the inadequacy of qualitative or quantitative methods alone to capture trends and details in the context of the research studies (Creswell and Creswell, 2018), so the strengths of one approach cover the weaknesses of the other, thus providing more evidence for studying the research study problem and helping to answer questions that neither quantitative nor qualitative methods alone can (Creswell and Plano Clark, 2018).

In the qualitative section, a design based on descriptive phenomenology was used (Moustakas, 1994; Giorgi, 2009), in which the researchers sought to describe the experiences lived by individuals for a specific phenomenon (Creswell and Creswell, 2018). In the quantitative section, a quasi-experimental design was used, in which there was no random assignment of participants to the experimental or control groups. This study was carried out with a pragmatic worldview, in which the focus was on the consequences of the research study and the question asked rather than the methods, thus employing multiple methods of data collection to inform the problems analyzed (Creswell and Plano Clark, 2018). This worldview is perfectly suited to the mixed methods design carried out in this work, and its philosophical assumptions are shown in Table 1.

**Table 1:** Philosophical assumptions of pragmatic worldview.

**Ontology**	**Epistemology**	**Axiology**	**Methodology**	**Rhetoric**
Singular and multiple realities	Practicality	Multiple stances	Combination	Formal or informal

### Sample

According to the coaches, students, and schools that showed interest in, and commitment to, participating in the research study, sample selection was convenient and non-probabilistic (Patton, 2015). A total of 30 girls participated in the experimental (*n* = 15, age = 8.93 ± 0.80) and control groups (*n* = 15, age = 8.80 ± 0.86) during the 2018/2019 school year. The coach for the experimental group was a 26-year-old man with experience of 4 years in managing volleyball youth teams. The coach for the control group was a 25-year-old woman with an experience of 5 years in coaching volleyball youth teams. Both coaches were graduates in Physical Activity and Sports Sciences and had a Level 2 Volleyball Coach qualification from the Andalusian Volleyball Federation.

Both schools competed in the U-10 category in the Catholic Schools Competition, organized by the Catholic Schools Association of Seville. Both groups were selected because of their homogeneity in terms of sport (volleyball), gender (women), age (between 8 and 9 years old), training structure (two training sessions/week, 1.5 h/session, plus the weekend game), and season expectations (qualifying for the final phase of the competition). The inclusion criteria were as follows: (a) participation in the extracurricular volleyball activity at their schools; (b) willingness to participate in the study; (c) written consent of the parents/guardians; (d) attendance for at least 85% of the sessions; and (e) adequate program implementation fidelity.

This study followed the ethical commitment guidelines defined by the Declaration of Helsinki regarding the consent, confidentiality, and anonymity of the participants through an agreement signed by the boards of the schools and the parents/guardians of the players. This study was also approved by the PhD Research Committee of the Pablo de Olavide University (internal code 2345-N-20).

### Variables

The dependent variables analyzed were the two dimensions of responsibility: PR, which is the responsibility to assume the own actions of an individual and act in consideration of, and respect for, the value of people and things, and SR, which is defined as sensitivity toward the feelings and needs of colleagues, respecting their rights and cooperating and working together to achieve goals and negotiating conflicts (Jiménez and Durán, 2004). Both the objective perception of the level of each of the dimensions and the subjective perception of skills development in these dimensions throughout the intervention were analyzed.

The independent variable was the TESPODEP program (Muñoz-Llerena et al., 2019), which is a hybridization of the TPSR and SE models, and was focused on the development of values such as leadership, decision-making, and PR and SR through team sports. The program, following the recommendations of Gordon (2009), used the general structure of the TPSR as a basis, adding the sport-specific elements that come from the SE, while using methodological strategies that arise from both the TPSR and SE (Table 2). A total of 37 sessions of 90 min plus 14 competition games were carried out, implementing sports content and a concrete responsibility level (Table 3). It must be considered that level 5 (transference) was implemented in all sessions of the program through the group reflection and self-evaluation, and this level was also the focus during games.

**Table 2:** Team Sports Positive Development Program (TESPODEP) characteristics.

**E**	**Description**	**S**
P	Educate participants to be players in the full meaning of the term, helping them to develop as competent, literate, enthusiastic, and responsible players, capable of leading the group and of making decisions that benefit the team.	TPSR/SE
MF	Embedding, transference, empowerment, and being relational with youngsters	TPSR
O	Develop volleyball-specific physical, technical, and tactical skills; participate at an appropriate level; share, be responsible, and lead the management of the athletic experiences; improve decision-making ability in sport; and apply the learned skills and abilities outside the program	TPSR/SE
RL	1- Establishing a positive group climate; 2- Participation and effort; 3 – Self-direction; 4- Help and leadership; 5- Transference	TPSR/SE
SS	Relational time, awareness talk, action time, group reflection, and self-evaluation	TPSR
SSE	Season, affiliation, formal competition, performance recording, festivity, and culmination event	SE
GM	Setting expectations, giving opportunities for success, fostering social interaction, setting roles and tasks, mastery-oriented learning, leadership, giving voice, role in the evaluation, respect model, and transference	TPSR
SM	Group control routines, guided practice, independent practice, conflict resolution, coach's portfolio, and team identity	SE

*E, Element; S, source; P, purpose; MF, methodological foundations; O, objectives; RL, responsibility level; SS, session structure; SSE, sport-specific element; GM, general methodological strategies; SM, specific methodological strategies*.

**Table 3:** Sessions, contents, and levels throughout the program.

**S**	**Content**	**RL**
1	Overhead pass-T; Underarm pass-T	I
C	Competition	5
2	Overhead pass-T; Underhand serve-Sec	1
3	Underarm pass-T; Underhand serve-Sec	1
C	Competition	5
4	Overhead pass-T+M; Underarm pass-T+M	1
5	Overhead pass-T+M; Underarm pass-T+M	1
6	Overhead pass-P+M; Underhand serve-Sec	2
7	Overhead pass-T+M; Underarm pass-T+M; Underhand serve-Sec	2
C	Competition	5
8	Overhead pass-P+M; Underarm pass-T+M; Underhand serve-Sec	2
C	Competition	5
9	Overhead pass-T; Underarm pass-T	1,2
10	Overhead pass-T; Underarm pass-T; Underhand serve-Sec	2
11	Overhead pass-T+M; Underarm pass-T+M	2
12	Overhead pass-T+M; Underarm pass-T+M; Underhand serve-Sec	2
C	Competition	5
C	Competition	5
13	Overhead pass-T+M; Underarm pass-T+M; Underhand serve-Sec	3
14	Overhead pass-T+M; Underarm pass-T+M; Underhand serve-Sec	3
C	Competition	5
15	Overhead pass-T+M; Underarm pass-T+M; Underhand serve-Sec	3
C	Competition	5
16	Overhead pass-P; Underarm pass-P; Underhand serve-Sec	3
17	Overhead pass-P; Underarm pass-P	3
18	Underarm pass-P; Underhand serve-Sec	3
19	Overhead pass-P; Underarm pass-P; Underhand serve-Sec	3
C	Competition	5
20	Overhead pass-P; Underarm pass-P; Underhand serve-Sec	3
21	Underarm pass-P; Underhand serve-Sec	3
C	Competition	5
22	Overhead pass-P; Underarm pass-P	3
23	Overhead pass-Setting; Underarm pass-P	3
24	Overhead pass-Setting; Underarm pass-P; Underhand serve-P	3
C	Competition	5
25	Overhead pass-Setting; Underarm pass-Receiving; Underhand serve-P	4
26	Overhead pass-Setting; Underarm pass-Receiving; Underhand serve-P	4
27	Overhead pass-Setting; Underarm pass-P	4
28	Overhead pass-Setting; Underarm pass-Receiving; Underhand serve-P	4
C	Competition	5
29	Overhead pass-Setting; Underarm pass-P	4
30	Underarm pass-Receiving; Underhand serve-P	4
31	Overhead pass-Setting; Underarm pass-P	4
32	Overhead pass-Setting; Underarm pass-Receiving; Underhand serve-P	4
C	Competition	5
33	Overhead pass-Setting+attack (jump); Underhand serve-P	4
34	Overhead pass-P; Underarm pass-P; Underhand serve-Sec	4
35	Overhead pass-P+attack (jump)	4
C	Competition	5
36	Underarm pass-Receiving+defense; Underhand serve-P	4
37	Overhead pass-P+attack (jump)	4

*RL, Responsibility level; I, introduction; S, session; T, technique; M, movement; Sec, security; P, precision; C, competition*.

A brief description of the characteristics of the TESPODEP program is included as Supplementary Material.

### Instruments and Measures

#### Semi-structured Interviews

Semi-structured interviews were chosen and elaborated *ad hoc* from the adaptation of other interviews used in interventions based on the TPSR model (Patton, 2015; Manzano-Sánchez and Valero-Valenzuela, 2019); the interview structure was designed to determine the perception of the players about their PR and SR skills.

The questions asked were divided into four differentiated blocks. The first block aimed to assess the previous experience of the players in the practice of volleyball (e.g., “Had you practiced volleyball before starting this year?”) and their participation in other extracurricular activities, athletic or not (e.g., “Do you do any other after-school activity besides volleyball?”). The second block was oriented toward the perception of the players of their skills development after participation in the intervention program (e.g., “Do you think that the training sessions have helped you to be more responsible in training and competitions? And in your life outside of sport? Why?”). The third block focused on determining the perception of the players about the intervention program itself (e.g., “What do you think about trying to take care of the group's positive climate?”). Finally, the fourth block focused on the perception of the players of the work of the coach (e.g., “Do you think the coach had a good relationship with the team? Have you felt comfortable with him/her?”). The full script of the interview is included as Supplementary Material.

#### Field Notes

Field notes were taken after each training session by the researcher, who also had the role of the coach in the team (full participation role). The structure of the field notes was elaborated *ad hoc* adapting a diary structure used in the previous research studies (Escartí et al., 2006), while aiming to recognize the implementation of the program in terms of objectives, contents, responsibility levels, and methodological strategies and documenting the perception of the researcher of PR and SR skills of the participants. A model for the field notes is included as Supplementary Material.

#### Personal and Social Responsibility Questionnaire

To assess the responsibility variable in its PR and SR dimensions, the Personal and Social Responsibility Questionnaire (PSRQ; Li et al., 2008) was used and validated in the Spanish context by Escartí et al. (2011). The questionnaire consists of 14 items, seven each for PR (e.g., “I propose goals for myself”) and SR (e.g., “Respect for others”). All items are answered through a 6-point Likert-type scale, ranging from 1, totally disagree, to 6, totally in agreement with the formulation of the question. Confirmatory factor analysis [$\chi_{76}^{2}$ = 161.36, *p* < 0.001, comparative fit index (CFI) = 0.91, root mean square error of approximation (RMSEA) = 0.06, goodness of fit index (GFI) = 0.89, adjusted goodness of fit index (AGFI) = 0.88, standardized root mean square residual (SRMR) = 0.06.] and internal consistency (= 0.85 SR; = 0.74 PR) indicate that the PSRQ is a useful instrument for assessing PR and SR levels and their evolution throughout the program.

### Procedure

For the sample selection, an informative meeting was held with the parents of all of the players in each school, explaining the characteristics of the program and the procedure to be followed for all groups (experimental and control). Subsequently, the same protocol was followed with the players and coaches from each group, with emphasis on the voluntary nature of participation. The program was implemented in groups where there were 10 or more interested players, the coach was willing to implement the program, and the parents agreed to carry it out.

The coach for the experimental group knew the fundamentals of the TPSR and SE programs and was the main person responsible for the design of the hybridization of the TESPODEP program. However, following the indications of Escartí et al. (2009, 2010b) and Fernández-Gavira et al. (2018), 1 month before the intervention he received recycling training on the theoretical foundations, objectives, and methodological aspects of both the TPSR and the SE by experts in these programs (research coauthors of this study). After the training period, the design of the intervention program was revised, with minor modifications. Throughout the intervention (once a month), the coach of the experimental group met with experts in the program to continue with the training and to analyze the fidelity of the implementation (Manzano-Sánchez and Valero-Valenzuela, 2019). The fidelity of the program implementation was considered adequate after analyzing it and triangulating the data obtained from the TARE 2.0 observation instrument (Escartí et al., 2015; Manzano-Sánchez and Valero-Valenzuela, 2019), which assessed methodological strategies, participant behavior, and the field notes.

The interviews were carried out in the final 2 weeks of training, once the program intervention had finished. The interviews were conducted with the players at the end of the session, without having established a time limit for each of them, which made it possible to collect between three and four interviews daily. The interview protocol was based on that proposed by Creswell and Creswell (2018). Interviews were recorded and transcribed verbatim. Field notes were completed daily after the training session, between 30 and 90 min after the end (depending on the availability of the researcher). All data were recorded in writing on a record sheet using the Microsoft Word (Office 365) software.

The PSRQ was completed before the beginning of the training sessions, under the supervision of the main researcher, ensuring the anonymity and sincerity of the responses, and answering any doubts that might arise during the process. Before starting, the instructions for correct completion of the PSRQ were explained and emphasis was placed on maintaining a relaxed atmosphere to promote concentration and maximum accuracy in the answers (Escartí et al., 2009, 2010a, 2011; Valero-Valenzuela et al., 2020a). The reliability in the pretest and posttest was 0.76 and 0.87 for SR and 0.83 and 0.86 for PR.

### Data Analysis

#### Interviews and Field Notes

**Figure 2:**
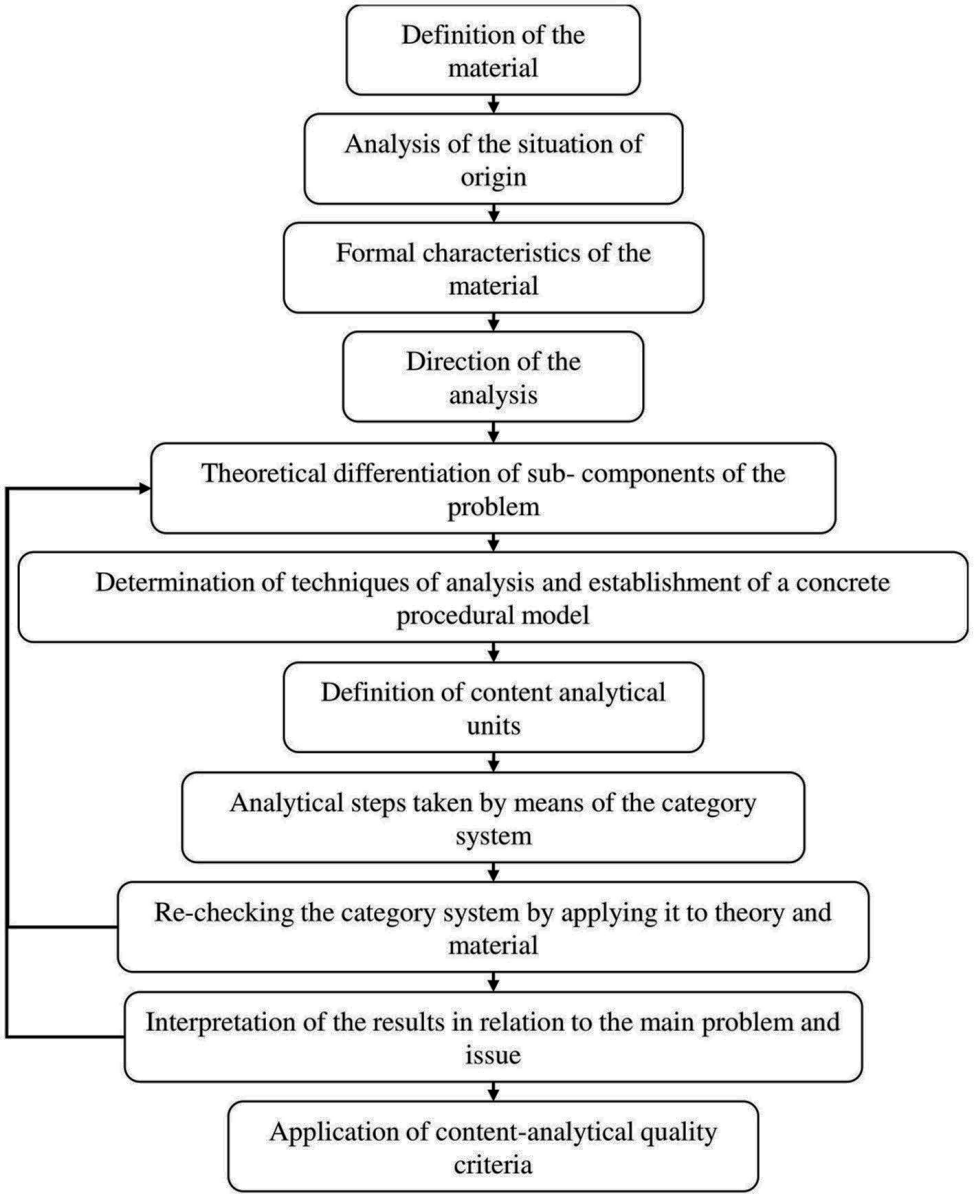
Qualitative content analysis procedure.

**Figure 3:**
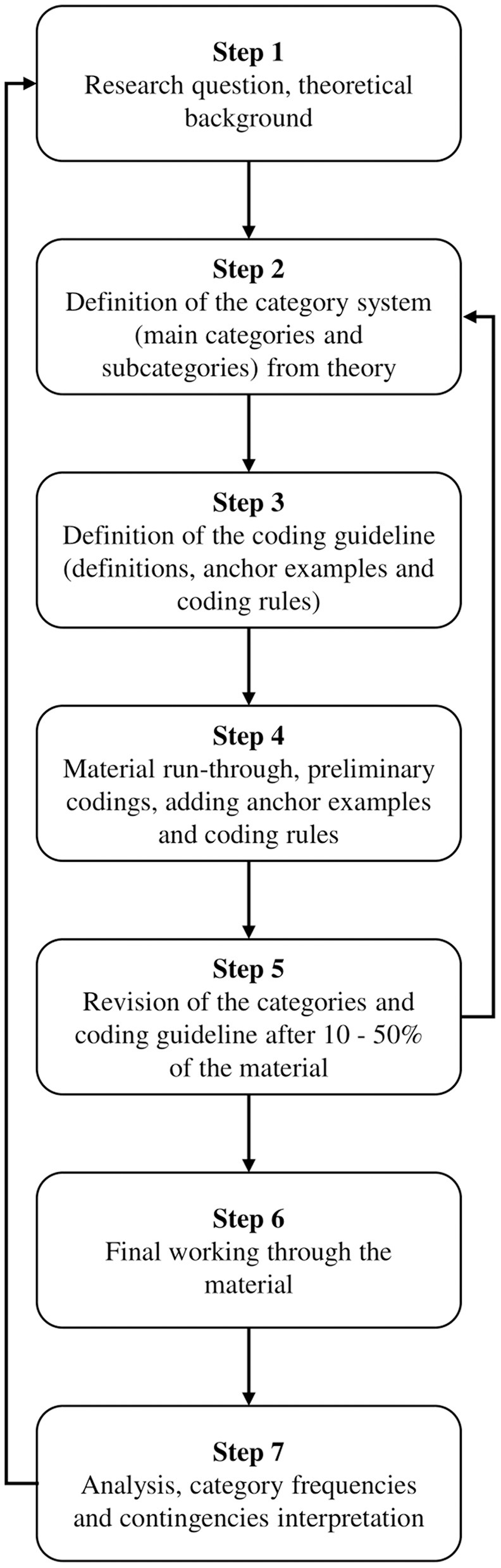
Steps taken in the deductive approach.

**Figure 4:**
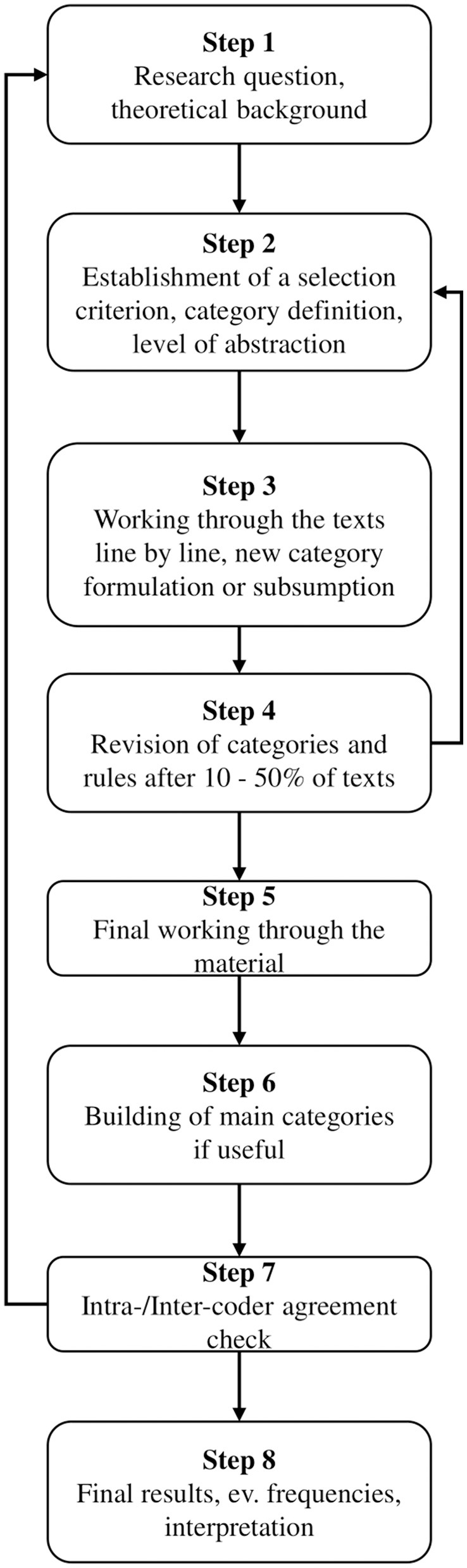
Steps taken in the inductive approach.

Qualitative content analysis (QCA) was the analysis strategy applied (Figure 2), utilizing the theme analysis technique, aiming to “retain the strengths of quantitative content analysis and against this background to develop techniques of systematic, qualitatively oriented text analysis” (Mayring, 2014, p. 39). Within the theme analysis technique of Mayring, a deductive approach was initially used to formulate the main categories of analysis based on the objectives of the study, semi-structured interview questions, and sections of the field diary (Figure 3); subsequently, the inductive approach was applied to formulate the secondary categories of analysis based on the emerging ideas of the participants about their perceptions on the development of PR and SR (Figure 4).

The quality criteria applied in the qualitative analysis were chosen based on the reflections of Smith and McGannon (2018). These criteria were as follows: (1) methodological integrity procedure (Levitt et al., 2017); (2) intervention of another researcher as a critical friend (Brewer and Sparkes, 2011); and (3) triangulation of analysts (inter-coder reliability) and sources (using multiple data sources) (Patton, 2015; Creswell and Creswell, 2018). Table 4 shows the unification of all of these criteria within the methodological integrity procedure proposed by Levitt et al. (2017). This analysis process was carried out using the qualitative analysis software NVivo™ 12 Plus.

**Table 4:** Methodological integrity.

**Fidelity**		**Utility**	
Adequate data	Triangulation was carried out, collecting data from two main sources (players and coach)	Contextualization of data	Information regarding the characteristics of the participants and researchers who have been part of the study has been presented
Perspective management in data collection	The originating paradigm has been explained, in addition to the second author acting as a “critical friend” to avoid distorting the data	Catalyst for insight	Interviews and field notes have been used as a form of data collection, after reflection by the authors on the best way to generate information that allows in-depth analysis
Perspective management in data analysis	Analyst triangulation was used to ensure the fidelity of the analyzed results	Meaningful contributions	The study has had a positive impact on the development of the participants in the variables analyzed, in addition to showing the scientific community the effectiveness of the implemented program
Groundedness	The results obtained were analyzed and discussed, making reference to quotations and reflections	Coherence among findings	The data obtained are integrated to show the relationship between the different results, including reflection on the congruencies and discrepancies

#### Personal and Social Responsibility Questionnaire

SPSS 21.0 software was used for the statistical analysis of the questionnaires and “psych” 1.4.2.3 of R 3.0.3 for the coefficient ω. For the PSRQ analysis, the main descriptive statistics were calculated, and normality tests were carried out. The asymmetry and kurtosis indicators of the variables were used for the univariate normality analyses, taking the limits of asymmetry and kurtosis in absolute values (Curran et al., 1996). Reliability analysis was carried out to check the internal consistency of the questionnaires. Cronbach's alpha (α) was used, equal to or> 0.70 (Nunnally, 1978; Nunnally and Bernstein, 1994), and the omega coefficient was used (ω; McDonald, 1999).

A repeated measures ANOVA was then carried out. A MANCOVA was used in the group factors (control group and experimental group) and two measurements (pretest and posttest), followed by the Bonferroni *post hoc* test to identify possible intragroup and intergroup differences and statistical power. Values of *p* < 0.05 and p <0.01 were used for statistical significance.

#### Integration

The joint display technique was used for data integration (Guetterman et al., 2015; Creswell and Creswell, 2018; Creswell and Plano Clark, 2018), combining the data in a table and making interpretations based on the table. Four key aspects were followed (Creswell and Plano Clark, 2018) to evaluate the quality of the research as a study that uses mixed methods: (1) The authors collected and analyzed both qualitative and quantitative data rigorously, taking into account research questions and hypotheses; (2) they then intentionally integrated the two types of data and their results; (3) these procedures were organized into specific research designs so that the study was developed in a logical way; and (4) these procedures were framed within theory and philosophy. The joint display was carried out using the Microsoft Excel (Office 365) software.

## Results

### Interviews

For the interview analysis, a first deductive coding was used on the basis of the existing studies and theory (Li et al., 2008; Escartí et al., 2011; Hellison, 2011) to determine the main categories of each dependent variable analyzed (PR and SR), establishing the categories of autonomy and effort (PR), and respect and caring and helping (SR). Once deductively encoded, inductive analysis of the coding was performed (Mayring, 2014). The coding frequencies for each category analyzed are shown in Table 5.

**Table 5:** Interview coding frequencies.

	**1. Personal responsibility**	**1.1. Self-direction**	**1.2. Effort**	**1.3. Responsible for the equipment**	**2. Social responsibility**	**2.1. Respect**	**2.2. Caring and helping**
Coding frequency	145	36	108	3	128	76	56

#### Personal Responsibility

Personal responsibility included the main categories of autonomy, effort, and responsibility for the equipment.

##### Autonomy

This category refers to the subjective perception of the player about her ability to be independent in tasks, without the need for coach supervision, and to set her own goals and develop an action plan to achieve them. This category included the secondary categories of self-confidence, goal setting, and autonomous work. *Self-confidence* is an abstract skill, and although several players considered that they had developed it, they nevertheless found it difficult to define why they thought they had improved. In particular, six players referred to having learned to trust themselves.

P04: “I (have learned to) set my own goals and trust myself.”

P07: “Because I don't forget things.”

In relation to *goal setting*, most of the players considered that they had acquired the ability to set goals, both in sport and in their daily lives, and that they were capable of acting to achieve those goals.

P03: “I now make more decisions than before, because before I didn't make any decisions, but now I say, well now I'm going to try to do this just because, because it turns out badly or (…) just because I want.”

P14: “I don't set myself a lot of goals either, but at least, but I have set the goal of cleaning my room every day.”

However, there were some players who considered that they had not been able to internalize the complete process or that they had learned it but were not able to carry it out on their own, as it depends on other circumstances such as mood.

P11: “It depends on what (the goal is). Because now I am a bit lazy.”

Six players referred to *autonomous work* in the interviews. Of these, four claimed to have learned to act and decide on their own throughout the program, while the other two denied having acquired this ability (although they considered that they were capable of acting on their own in aspects relative to other categories).

P03: “It was very good for me, because in addition to the fact that I could do what I felt would be better—in my life apart from volleyball as well—I have also said: well, look, this can be better for others, so I'm going to do it.”

P06: “Yes, I am (more) responsible. I make the bed, I set the table, I brush my teeth.”

##### Effort

This category refers to the subjective perception of the player that she experiences the content of the program in a positive way, developing self-motivation and taking responsibility on her own, participating in the tasks/games proposed, trying to do the best in the games in which they participate, and conceiving success in terms of participation, improvement, and mastery, depending mainly on their own effort. The secondary categories of effort in tasks, motivation, and participation were included in this category.

Regarding *effort in tasks*, practically all of the players affirmed that they believed they had learned to try harder in the athletic context, although one of them believed that she did not always make the best effort and only did it halfway, while another two participants only felt they achieved this when they did not “act like a dummy.”

P15: “I make an effort in training. (…) I am already trying my best. I can't try harder.”

P14: “(I make an effort) but sometimes (…) in training, I don't know why, I start to act like a dummy.”

P09: “Yes. Well, only if I don't act like a dummy.”

P15: “In other words, effort—because I already make more of an effort (…) in games, I no longer treat it like a joke, because before I treated it like a joke.”

P02: “My favorite game is Claret's second, because I passed a serve. And (in that game) I tried harder than in training.”

Outside the athletic context, most of them considered that they had also learned to try harder, mainly in the school and family environments.

P14: “Yes, because now I do my homework every day—I study every day.”

P02: “(Refers to her improvement in) Obeying my parents, studying….”

Regarding *motivation*, 10 players noted that they had learned to motivate themselves when carrying out a task or playing a game. Of these, two believed that they were capable of self-motivation only sometimes.

P14: “I have learned to motivate myself more. (…) I was (sad) before… and now, I'm happy all the time.”

P01: “I do (motivate myself). Because in the game that we played, that I didn't want to go to, I went. And I played well.”

P06: “I have to do it because I have (…) to achieve it. Myself.”

Regarding *participation*, again 10 players affirmed that they had learned to participate more in tasks and games than before the program.

P10: “Yes, I didn't (participate) before.”

P03: “(I have learned) not to stand still but to try (to participate). (…) Now I participate in everything. (…). (The program) Has also helped me to participate in everything in my life.”

P15: “And participation, well. I try to participate in everything.”

P06: “(I have learned to) Participate in all games.”

In contrast, only one player believed that she had not learned to participate more in tasks and activities than before participating in the program (altogether with the effort, as seen previously). The rest of the players had nothing to say on the matter.

P11: “That just when we started level 2 (of the program), which was participation and effort, just on those days I neither made any effort nor participated because it was very hot. (…) When I'm hot I don't really want to learn.”

##### Responsible for the Equipment

This category refers to the subjective perception of responsibility related to the treatment, care, and maintenance of the training equipment. Only two players referred to this category in the interviews. They had the perception that they had not learned to be fully responsible because they were not able to take care of their own equipment and, sometimes, the training equipment itself.

P02: “No, because I lose all of the sweatshirts. Have you seen me?”

P04: “First, (…) because I lose my sweatshirt a lot. And second, because when we are playing, when we are playing and all that in couples, I lose the ball. A lot of balls start to fall, okay, and now I hit another, I don't know what, and I'm not responsible for the ball. And then, with my backpack, I always forget my backpack, and that doesn't help me.”

#### Social Responsibility

The main categories of respect and caring and helping were found in the SR perspective.

##### Respect

This main category refers to the subjective perception that the rights and feelings of others are respected, controlling the attitude and behavior of an individual in such a way that the rights and feelings of others are respected, resolving conflicts peacefully, and including everyone in the group in the tasks/games. This category included the secondary categories of cohabitation rules, interpersonal relationships, and conflict resolution.

Regarding *cohabitation rules*, most of the players referred to the fact that they had learned to respect and follow the rules of coexistence that were established at the beginning of the season by mutual consent of all team members.

P04: “(I have learned to) Respect the rules.”

P07: “I have learned to… respect the rules… and that's it.”

P05: “That next time I'm going to bring chewing gum, but for after training.”

Some of them also showed an improvement in following the rules outside the team context, in the family environment.

P02: “(Referring to her improvement) Obeying my parents, studying…”

P05: “The yes (referring to what she has learned) is that I obey my parents more.”

Regarding *interpersonal relationships*, several of the players referred to the fact that they had learned not to interrupt and to pay attention to the coach (either the main coach or the one with the role of coach). However, there were only a couple of players who admitted that they did not always pay attention to them.

P10: “(I have learned) To pay attention to the teacher.”

P11: “That sometimes I listen to you, but other times I don't.”

Some of the players also talked about how they applied what they had learned to their lives outside of volleyball, mainly in their family relationships. However, most of them believed that they had not become more responsible, as they kept fighting with their siblings.

P05: “Yes and no (I have learned). Because I keep bugging my brother. (Although already) I don't hit my brother. But if he hits me, I hit him.”

P13: “Yes (I have learned), because I don't fight so much with my sister anymore.”

P08: “Me sometimes, less than before. (But) No (I haven't learned). Because I keep hitting my sister.”

In relation to *conflict resolution*, practically all of the players had learned to resolve conflicts peacefully. In the interviews, reference was made both to the learning of conflict resolution itself and to the knowledge of the specific steps to resolve conflicts, so participants were able to resolve them autonomously.

P14: “I think that I have learned to resolve conflicts. (…) Because now I get a bit into the fights of others, but now I at least resolve it, more or less.”

P15: “Because you have taught us to resolve conflicts with the… with the list you gave us. (…) I know, now, that if we have a conflict, then we tell you ‘We are going to solve the conflict,’ and we solve it …”

P13: “Well, I've also learned to resolve conflicts, because (…) I didn't know before.”

P08: “When there is a conflict with someone—knowing the steps.”

There were also players who had begun to apply what they had learned about conflict resolution in other contexts outside the team, or they were able to resolve conflicts between other people, although one player thought that she had not learned anything in this regard.

P03: “I have solved many conflicts (…), I get involved in everything. I have to know everything.”

P04: “(Outside of training I have learned) To pay more attention to others, to try to resolve more conflicts, to play more volleyball, and to help others when they are alone or something, to try to improve what happens to them.”

P11: “That I have resolved a lot of conflicts at school.”

P03: “Yes. I have solved a lot of problems. Too many. I mean, once, one day, everyone was crying. (…) And I went one by one trying to console them (…).”

P07: “For example, P09 gets angry with P15. Then I go and tell them what happened, they tell me and (I help them solve it).”

P14: “I don't. Not a single one. (…) What I did is tell them (the crying children) “cry at home all you want, be happy here,” and that's it.”

##### Caring and Helping

This category refers to the subjective perception of the player that the needs and feelings of others are recognized and that they are capable of putting themselves in the place of the other in an empathic and compassionate way, listening and responding without judging, helping without being arrogant, and understanding the importance of helping only when the other wants that help. This category includes helping others and effective interpersonal communication as secondary categories.

Regarding the secondary category *helping others*, most of the players claimed that they had learned to help other people when they needed it, to a greater or lesser extent. While some players found it more difficult than the others, most of them had adequately learned to help others.

P10: “Yes (I am able to help my teammates when they get blocked), but I laugh a little.”

P04: “(I have learned) How to treat people, more or less in the ways that should or should not be—how you have to behave and how you should not behave and help others. (…) (And outside the program) To pay more attention to others, to try to resolve more conflicts, to play more volleyball, and to help others when they are alone or something, to try to improve what happens to them.”

P11: “Help children who cannot (do something).”

P15: “What I (…) liked the most it's when we… taught, those of last year, to those who did not know, who had started, to help them learn the finger and forearm touch.”

Several of the interviewees specifically described situations in which they had transferred this skill to their daily lives, mainly in helping at home or helping classmates or friends.

P03: “I've never tidied up my room, unless my mother tells me off for tidying it up. (…) This weekend I got up and said come on, I'm going to take some work off my mother, and I make the bed. Then I take a shower and say well, I'm going to throw all the clothes… into the laundry.”

P14: “I… I started last week, because I said I'm going to make my mother happy and tidy up the room a little. Because everything was lying around (…) and what do I do? Well, I tidied it up.”

Regarding *effective interpersonal communication*, approximately half of the players stated that they had developed their ability to communicate during the program, some referring to activities carried out that helped them improve their interpersonal communication, and others referring to the loss of shyness thanks to the program.

P03: “(I have learned) To express myself. Before I was very shy. Now they call me a rascal (not shy).”

P14: “I have learned to talk more with people. Before I was super shy and now I talk more.”

P06: “The… (communication). (…) We had minigames, too. (…) That we had to solve together to have more communication between us.”

Several of the players expressed their learning and development in terms of behavior and treating others empathically and with compassion.

P02: “I have learned how to behave and how I have to treat (…) people.”

P04: “(I have learned) How to treat people, more or less in the ways that can or cannot be, how you have to behave and how you should not behave and help others.”

P13: “I have learned to be more assertive.”

P05: “(I've learned to) Get less and less angry. (…) To be assertive with others.”

### Field Notes

The same process as with the interviews was followed for the analysis of the field notes, starting from a first deductive coding, establishing the main categories of autonomy and effort (PR) and respect and caring and helping (SR), and subsequently applying inductive analysis. The coding frequencies with respect to each category analyzed are shown in Table 6.

**Table 6:** Field note coding frequencies.

	**1. Personal responsibility**	**1.1. Self-direction**	**1.2. Effort**	**2. Social responsibility**	**2.1. Respect**	**2.2. Caring and helping**
Coding frequency	38	11	27	38	17	23

#### Personal Responsibility

The main categories of autonomy and effort were also found in PR.

##### Autonomy

This main category refers to the subjective perception of the coach of the ability of the players to be autonomous in tasks, without the need for coach supervision, and the establishment of their own goals and the development of an action plan to achieve them. The secondary categories of goal setting and autonomous work were included in this category.

Regarding *goal setting*, the perception of the coach was that, during training, in level 3 of the program (autonomy), the group had reflected on the usefulness that goal setting can have in the daily lives of the players and the need to decide their own goals to achieve during the next month of training.

“In the final reflection, the usefulness of goal setting in different areas of life is explained to the players, and they are made to think (…) about a volleyball goal and another non-athletic goal that they want to achieve during next month, to focus on the training sessions to gradually reach them.” (12th session)

The group progressed gradually during level 3 in its goal setting and learned to develop an action plan to achieve goals, dedicating numerous reflections during the training sessions to encouraging reflection and the acquisition of these skills.

“In addition, they are looking together for a way to achieve the objectives that the players set themselves in the previous session.” (13th session)

“In the final reflection, how to use the ‘checklist’ is explained, as well as how important it is to develop one when setting a life goal, taking each of the elements that appear in it seriously.” (14th session)

Regarding *autonomous work*, one of the points highlighted by the coach was the development of the autonomous work capacity of the players, which was chaotic at the beginning of level 3 (they were not able to train on their own without an attentive coach), but by the end, the coach perceived that the group was already capable of functioning on its own, without the need for a coach and without any danger of stopping the training due to the absence of the player with the role of coach.

“In the final reflection, the conclusion is reached that, if at a specific moment the coaches have to attend to urgent things, the group cannot stop training and start to fall into chaos in training.” (13th session)

“Last session of level 3, this seems to be already mastered and I see players with the ability to function autonomously.” (23rd session)

##### Effort

This category refers to the subjective perception of the coach of whether the players experienced the program content in a positive way, developed self-motivation and took responsibility for their own behavior, participated in the tasks/games proposed, tried to do their best in each task or game in which they participated, and conceived success in terms of participation, improvement, and mastery in a certain task, depending mainly on their own effort. The secondary categories of training, roles, and transference were included in this category.

Regarding *effort in training*, although it was worked on more consciously and intentionally in level 2 (participation and effort), it was a regular element of discussion throughout the program for the coach. The perception of the coach indicated that it was usual for the group to train properly in terms of effort, with the players experiencing the sessions positively and showing adequate effort and predisposition to do the tasks (as the program progressed, the level of effort and participation increased).

“In the final reflection, the conclusion is reached that in this session there has been progress within level 2, and they have done their part (both players and coaches) to carry out the tasks with good participation and attitude.” (6th session)

“In general terms, the session developed satisfactorily, with the players engaging at a good level, except for a period of 5 min at the beginning of the second task, where there are several of them that are not sure of the groups that they have to make and they do not work on the task.” (18th session)

However, the coach indicated that there were sessions in which the effort presented by the players left a lot to be desired and did not allow the development of proper training, or they simply did not make their best effort.

“The players start the session well (warm-up and task 1), but the moment we advance to task 2 (somewhat more complex), they totally disconnect and each one begins to go on their own terms, to talk and stop training. I try to make them aware of the importance of level 2 when it comes to improving in sport and some of them get more involved, but others go their own way.” (7th session)

The coach highlighted that, throughout the intervention, many reflections were made so that the players understood effort as something necessary for the proper development of both the training as a whole and themselves as players.

“In the final reflection, they came to the conclusion of the importance of the coaches being aware of the group, correcting and helping the others, in addition to having, all of them, a feeling of being able to make a serious effort if they set out to do so.” (9th session)

“In the final reflection, I try to make them understand that if they do not train well there is no improvement, in addition to understanding how I feel as a coach when all my effort to prepare a productive session goes to waste because they don't want to work or train.” (28th session)

Regarding *roles*, the coach referred to the effort that the players presented with respect to their own roles within the group in the training sessions. On most occasions, the effort made by a specific role was highlighted in a positive way or the improvement in her performance (due to greater effort on the part of the player) from one session to another.

“The coach (…) is not able to control the group and it is difficult for her to explain the tasks and for the others to understand them, but she puts in all her effort to achieve it.” (3rd session)

“In the final reflection, (…) in this session there was progress within level 2, and they have done their part (both players and coaches) to carry out the tasks with good participation and attitude.” (6th session)

Finally, the coach also made the players think about the importance of showing a high effort in training by the players with roles, especially those with greater responsibility.

“In the final reflection, a conclusion is reached about the importance of the coaches being aware of the group, correcting and helping others, in addition to having—all of them—a feeling of being able to make a serious effort if they decide to do so.” (9th session)

Regarding *transference*, throughout the program, the coach tried to foster the learning of the player about the importance of effort in other areas of their lives through reflection, and not only in the sports context, perceiving that they were integrating this message and carrying out actions in their day-to-day activities where they have to strive.

“In the final reflection, I lecture them on their attitude in the second half of the training, then highlighting the things they have done well throughout the training and the benefits they can get from working hard and participating to the best of their ability. The team mostly agrees and comes to the same conclusion (…).” (7th session)

“They are made to think about the usefulness of this capacity for effort in other areas of life, giving them examples.” (9th session)

#### Social Responsibility

The main categories of respect and caring and helping were found in SR.

##### Respect

This category refers to the subjective perception of the coach that players respect the rights and feelings of others, controlling their own attitudes and behaviors such that the rights and feelings of others are respected, resolving conflicts peacefully, and including everyone in the group in the tasks/games. The secondary categories of respect for others, respect for the decisions of the coach, and conflict resolution were included in this category.

Regarding *respect for others*, the coach stated that, although in general terms the players had maintained a high level of respect for others, sometimes this respect was conspicuous by its absence. At times, this resulted in not respecting the cohabitation rules set out in the group at the beginning of the season, which were chosen democratically, albeit in the first sessions they had not yet internalized the rules and it is normal that they were not capable of respecting all of them.

“At the level of values within level 1, there is a good training environment, although not all the rules of coexistence are respected.” (1st session)

“The first part of the session proceeds normally, while in the second part I allow 5 min to drink water and the players arrive 15 min later, thus losing almost a quarter of the training time.” (37th [last] session)

There were also occasions when the players did not respect the person in charge of the group, interrupting while (s)he spoke or not doing what they were told, especially in the first three levels of the program.

“In the final reflection, the players are a bit agitated and are not able to remain calm and silent, interrupting me many times while I try to speak.” (18th session)

“In the final reflection, I ask them if it really compensates them for all the time they lost and wasted in speaking and not doing what the coaches asked, in addition to having a negative impact on not carrying out the final reduced/real game task.” (22nd session)

In relation to *respect for the decisions of the coach*, the general perception of the coach was that the group adequately respected the decisions of the coach (player who has assumed the role of coach), although in the first sessions of the intervention they still did not grasp the concept that they were self-training and thus did not fully respect the instructions of the fellow trainers. As the program progressed, this aspect improved.

“The group does not entirely respect the coach's instructions, and they barely make any self-criticisms in the personal reflection.” (1st session)

“In the final reflection, I ask them if it really compensates them for all the time they lost and wasted in speaking and not doing what the coaches asked, in addition to having a negative impact on not carrying out the final reduced/real game task.” (22nd session)

Regarding *conflict resolution*, the coach referred to his perception that the group acquired and applied conflict resolution in training, all during level 1 of the program. Some references indicated that the players were learning and trying to apply the proposed conflict resolution mechanisms, and others exposed concrete cases in which some players (with responsible roles) applied them to solve problems among other group members. However, some performed better than others.

“She has been able to resolve a conflict between three players autonomously, demonstrating her development in leadership, decision-making, and conflict resolution skills.” (2nd session)

“(Referring to the 2nd coach) Good role in conflict resolution.” (30th session)

“Trying to apply learned conflict resolution mechanisms when one arises.” (1st session)

“In the final reflection, conclusions are drawn about what has been learned, about how what has been learned can be applied when resolving conflicts autonomously outside of training, and occasional self-criticism of the leading players in the group begins to appear.” (4th session)

##### Caring and Helping

This main category refers to the subjective perception of the coach that the players recognize the needs and feelings of others and are able to put themselves in the place of another in an empathic and compassionate way, listen and respond without judging the other, helping without being arrogant, and understand the importance of helping only when the other wants that help. This category includes the secondary categories of helping others and effective interpersonal communication.

Regarding *helping others*, the coach considered that the players were able to internalize the importance of helping others when they needed it, highlighting the help that the player with the role of coach gave the rest of the group when she directed them.

“In the final reflection, a conclusion is reached about the importance of the coaches being aware of the group, correcting and helping the others, in addition to all of them having a feeling of being able to make a serious effort if they decide to do so.” (9th session)

However, one of the players noted a lack of SR in this sense, because she left the coach alone (being 2nd coach) in one of the sessions to go to a birthday party.

“The second coach (…) did not go to training because she has a birthday, which indicates a great lack of commitment and respect for her teammates.” (25th session)

In relation to *effective interpersonal communication*, the perception of the coach was that the group seemed to have acquired knowledge about the concept of effective interpersonal communication, what it is, what it is for, or why it is necessary to learn it.

“In the final reflection, I emphasize the need—both when they are coaches and when they need to communicate in their day to day—to know how to communicate effectively.” (24th session)

“In the final reflection we talked about the importance of styles when leading groups or communicating outside of sports, in their personal lives.” (37th session)

It was observed that learning took place gradually throughout the application of level 4. At the beginning of the level, the girls did not control interpersonal communication at all; at the end of the program, they were able to communicate assertively while respecting the turn of each other.

“This time they do begin to ask to speak and to speak when it is their turn, although sometimes I have to remind them and ignore them if they don't.” (29th session)

“In the final reflection, they are asked about their experience with the different styles, and which style they believe is the most important when dealing with other people, reaching the conclusion that it is the assertive style that best suits effective and adequate communication.” (35th session)

### Personal and Social Responsibility Questionnaire

The results obtained in the Kolmogorov-Smirnov normality test and the homogeneity of variances (Levene's test) showed a normal distribution of the data. The results referring to the descriptive analysis of the instruments used in the study were taken at two different moments (pretest and post-test; see Table 7). In the reliability test, some items had to be eliminated (α > 0.70) so that the PR-Pre and SR-Post factors presented adequate reliability values (α > 0.70; ω > 0.70).

**Table 7:** Descriptive statistics and reliability analysis.

**Variable**	**M**		**SD**		**α-Pre**	**α-Post**	**ω-Pre**	**ω-Post**
	**Pre**	**Post**	**Pre**	**Post**				
PR	5.13	5.30	1.02	0.70	0.66	0.70	0.73	0.73
SR	5.24	5.15	0.54	0.74	0.70	0.60	0.80	0.71

*M, Mean; SD, standard deviation*.

In the intergroup analysis, no significant differences were observed between groups in the PR and SR variables, the magnitude of the effect size being trivial (η*2* < 0.20). In the intragroup analysis, no significant improvements were observed in any group (Table 8).

**Table 8:** Intergroup and intragroup analysis.

**Variable**	**Group**	**Pre**			**Post**		
		**M**	**SD**	**p**	**M**	**SD**	**p**
PR	Experimental Control Comparative	5.13 5.13 5.13	1.10 0.97 1.02	0.33	5.48 5.13 5.30	0.63 0.76 0.70	0.18
SR	Experimental Control Comparative	5.33 5.16 5.24	0.68 0.37 0.54	0.89	5.07 5.24 5.15	0.90 0.57 0.74	0.52

*M, Mean; SD, standard deviation*.

### Integration

The aim of the following integration was to develop integrated results and interpretations that could provide a more comprehensive understanding of the results analyzed and to validate and confirm the qualitative and quantitative results. Table 9 shows the integration of results through a joint display.

**Table 9:** Integration.

**V**		**PR**			**SR**	
**PSRQ**		**Pre 5.13 ± 1.02**	**Post 5.30 ± 0.70**	**Sig. 0.37**	**Pre 5.24 ± 0.54**	**Post 5.15 ± 0.74 Sig. 042**
**MT**		**Self-direction**	**Effort**	**Responsible for the equipment**	**Respect**	**Caring and helping**
I	Cg	P11: “It depends on what (the goal is). Because now I am a bit lazy.”	P11: “That just when we started level 2 (of the program), which was participation and effort, just on those days I neither made any effort nor participated because it was very hot. (…) When I'm hot I don't really want to learn.”	P04: “First, (…) because I lose my sweatshirt a lot. And second, because when we are playing, when we are playing and all that in couples, I lose the ball. A lot of balls start to fall, okay, and now I hit another, I don't know what, and I'm not responsible for the ball. And then, with my backpack, I always forget my backpack, and that doesn't help me.”	P05: “Yes and no (I have learned). Because I keep bugging my brother. (Although already) I don't hit my brother. But if he hits me, I hit him.”	No data available
	Ds	P03: “It was very good for me, because in addition to the fact that I could do what I felt would be better—in my life apart from volleyball as well—I have also said: well, look, this can be better for others, so I'm going to do it.”	P15: “In other words, effort—because I already make more of an effort (…) in games, I no longer treat it like a joke, because before I treated it like a joke. (…) And participation, well… I try to participate in everything.”	No data available	P15: “Because you have taught us to resolve conflicts with the… with the list you gave us. (…) I know, now, that if we have a conflict, then we tell you “We are going to solve the conflict,” and we solve it …”	P04: “(I have learned) How to treat people, more or less in the ways that should or should not be—how you have to behave and how you should not behave and help others. (…) (And outside the program) To pay more attention to others, to try to resolve more conflicts, to play more volleyball, and to help others when they are alone or something, to try to improve what happens to them.”
FN	Cg	“In the final reflection, the conclusion is reached that, if at a specific moment the coaches have to attend to urgent things, the group cannot stop training and start to fall into chaos in training.” 13th session	“The players start the session well (warm-up and task 1), but the moment we advance to task 2 (somewhat more complex), they totally disconnect and each one begins to go on their own terms, to talk and stop training. I try to make them aware of the importance of level 2 when it comes to improving in sport and some of them get more involved, but others go their own way.” 7th session	No data available	“The group does not entirely respect the coach's instructions, and they barely make any self-criticisms in the personal reflection.” 1st session	“The second coach (…) did not go to training because she has a birthday, which indicates a great lack of commitment and respect for her teammates.” 25th session
	Ds	“Last session of level 3, this seems to be already mastered and I see players with the ability to function autonomously.” 23rd session	“In the final reflection, the conclusion is reached that in this session there has been progress within level 2, and they have done their part (both players and coaches) to carry out the tasks with good participation and attitude.” 6th session	No data available	“She has been able to resolve a conflict between three players autonomously, demonstrating her development in leadership, decision-making, and conflict resolution skills.” 2nd session	“In the final reflection, they are asked about their experience with the different styles, and which style they believe is the most important when dealing with other people, reaching the conclusion that it is the assertive style that best suits effective and adequate communication.” 35th session

*V, Variable; MT, main categories; I, interviews; FN, field notes; Cg, congruent; Ds, discrepant; Sig, significance*.

## Discussion

The main objective of this study was to analyze the effects of an intervention based on the hybrid TESPODEP program on the PR and SR of youth girl volleyball players. The results obtained showed similarities with other research studies that exhibited the benefits that can be achieved in PR and SR and their different dimensions (autonomy, participation and effort, respect, and caring and helping) thanks to the implementation of TPSR, SE, or a hybridization of the two (Meroño et al., 2015; González-Víllora et al., 2019; Manzano-Sánchez and Valero-Valenzuela, 2019; Manzano-Sánchez et al., 2019; García-García et al., 2020; Valero-Valenzuela et al., 2020a).

### Perceptions of the Players and the Coach About PR and SR Development

Concerning the perception of the players, practically all of them managed to develop their PR throughout the implementation. The greatest learning occurred in the effort applied to tasks and in autonomous goal setting, such that the majority perceived being able to try harder, participate more, set their own objectives and establish an action plan to achieve them, and motivate themselves in everything they do. At least half of them believed they had also learned to act autonomously and to trust themselves. These results are in line with those presented by other studies carried out with school-age participants (Stran et al., 2012; Meroño et al., 2015; Fernández-Río and Menéndez-Santurio, 2017; Antón-Candanedo and Fernández-Río, 2018) and might be caused by the methodological structure of the TESPODEP, which encourages autonomy and effort through the establishment of responsibility roles in the players, thus producing this personal development (Muñoz-Llerena et al., 2019).

In terms of SR, it seems that practically all of them developed skills related to this dimension. The greatest perceived development was shown in conflict resolution, in respect for cohabitation rules, and in helping others, capacities that are mostly considered to be capable of transferring to other contexts. To a lesser extent, several participants also improved their knowledge about interpersonal communication and relationships. These results could be attributed to the implicit characteristics of the TPSR program, because, through various aspects such as group and personal reflections or the integration of the content of responsibility in the tasks, this type of social development was promoted (Hastie and Buchanan, 2000; Gutiérrez et al., 2014; Menéndez-Santurio and Fernández-Río, 2016b, 2017; Fernández-Río and Menéndez-Santurio, 2017).

Regarding the perception of the coach, the results are in line with the insights of the players. As the intervention progressed, positive learning was observed both in PR (through the fostering of the capacity for effort, goal setting and action plans, being autonomous in tasks, and being able to train without exhaustive supervision of those responsible for the group) and SR (developing their skills of respect for others, resolving conflicts autonomously, interpersonal communication, and ability to help others and learn the importance of respecting the feelings and rights of others, the cohabitation rules, and the decisions of the group leader).

From the perception of the coach, the internalization of content and prosocial skills through group and personal reflections and self-evaluations carried out at the end of the sessions seem to be a key aspect in the acquisition of these lessons. Results in line with these perceptions have been presented in previous research studies and have been related to the perception of the teacher of the usefulness of PYD programs (Casey and Dyson, 2009; Gutiérrez et al., 2014; Manzano-Sánchez and Valero-Valenzuela, 2019) and their effect on the increase in PR and SR (Fernández-Río and Menéndez-Santurio, 2017; Menéndez-Santurio and Fernández-Río, 2017). The importance of self-reflection and critical thinking to interiorize learning has also been highlighted in the study (Stran et al., 2012).

One of the fundamental aspects that can be deduced from this study is the achievement of transference by the participants. They seem to be capable of transferring those lessons mentioned above into their daily lives. This transference is an essential characteristic of PYD programs that use sports as a means to achieve this skill transfer (Fraser-Thomas et al., 2005; Hellison et al., 2008; Hellison, 2011; Turnnidge et al., 2014; Chinkov and Holt, 2016; Whitley et al., 2016; Weiss et al., 2020). It is possible that the nature of volleyball (team sport, cooperative, etc.), together with the structure of the task assignments used based on the SE model and the methodological structuring of the TPSR, made possible the achievement of good levels of transfer in these dimensions of responsibility.

However, not all perceptions were positive. Within the PR variable, reference was made to negative or incomplete aspects of learning with respect to goal setting, autonomy, effort, participation, responsibility for the equipment, and the performance of roles. In SR, negative perceptions of learning are indicated when resolving differences between others, in interpersonal relationships, in helping others, and in respect for others. This lack of development in PR and SR, although it differs from what was previously stated, also occurred in a study based on implementations of pedagogical models in school-age participants (Gutiérrez et al., 2014). However, these considerations must be interpreted with caution, because most of them refer to specific players and situations, and not to the group as a whole. Therefore, it would be more appropriate to affirm that not all of the players had learned everything that the program can offer. Other individual social or psychological traits, as well as previous experiences practicing the sport, could have had an influence on these results.

### Integrating the Data: How Personal Perceptions Relate to Statistical Results

Regarding the PSRQ, the results indicate that the intergroup (experimental and control) and intragroup (experimental) differences are not significant, and therefore, it is understood that there were no differences within or between the two groups in PR and SR when comparing before and after, although the α and ω values obtained are sufficient to accept the reliability of the instrument under the conditions used in this study (Nunnally, 1978; Campo-Arias and Oviedo, 2008). These results differ from those shown in the study (Menéndez-Santurio and Fernández-Río, 2016b; Escartí et al., 2018; Manzano-Sánchez and Valero-Valenzuela, 2019; Manzano-Sánchez et al., 2019; Valero-Valenzuela et al., 2020a), perhaps because, as a general rule, published studies always present statistically significant results. This non-significance in the data could be due to the small sample size, which might not be large enough to allow the appearance of differences between and within the groups.

In data integration, because no significant positive results were reported in the PSRQ, the results obtained at the quantitative and qualitative levels differ to a great extent. Although there are some congruent results, because not all of the players perceived improvements in all of the analyzed aspects of PR and SR, nor did the coach attribute significant development to all categories, we must be cautious in drawing conclusions due to the relativity of the results: The majority of the group seem to have developed their PR and SR, but not all of the players have developed all of the capabilities that PR and SR comprise, and not all of them are capable of using them in all the situations that might arise.

Ensuring that the integrated data are 100% congruent is a complex task (O'Cathain et al., 2007; Slonim-Nevo and Nevo, 2009). In this study, we started from a more general conception of the dimensions of PR and SR (PSRQ), and those dimensions were compared with participant learning in much more concrete and deeper aspects within those dimensions (main and secondary categories in interviews and field notes), which makes it difficult to achieve a majority of congruent results in the integration, especially if they differ so much. In this respect, the authors considered that there may be greater confidence in the results obtained at a qualitative level, because the small sample used may lead to the absence of significant results in the PSRQ, following what has been carried out in other investigations that used convergent mixed methods (Creswell and Plano Clark, 2018).

The data integration was carried out following the criteria to ensure the trustworthiness of the study. Following Creswell and Plano Clark (2018), it can be stated that both the qualitative and quantitative data were collected and analyzed rigorously, taking into account the research questions and hypotheses, because data of both types were collected based on the objectives and hypotheses raised in the study, following adequate procedures for their thematic and statistical analysis, respectively. The two types of data and their results were intentionally integrated, in line with the stated reasons for choosing a convergent mixed methods design for the study and integration of the results obtained through a joint display to later discuss the congruencies and discrepancies of the results and then draw a conclusion about them. These procedures were organized into specific research designs so that the study was undertaken in a logical way, and all elements fit together logically and consistently. These procedures were also framed within theory and philosophy, appropriately marrying the pragmatic paradigm and the PYD theory on which the study was based with the mixed methods designs used. These criteria have been taken into account in multiple studies that use mixed methods to ensure their reliability (Ames et al., 2009; Craig et al., 2019; Vázquez-Diz et al., 2019; Leiter et al., 2020; Valero-Valenzuela et al., 2020a; Weiss et al., 2020).

### Theoretical and Practical Implications

This study helps to fill the gap in the study related to the effects of SBPYD programs in competitive settings by designing a hybrid program that could serve as a reference in team sports interventions based on PYD. Another strength of this research study is its contribution to the mixed methods research field, which is not widely used in competitive sports interventions.

From a practical view, this study may provide strategies for coaches and teachers in the design and implementation of training exercises that aim to enhance decision-making, leadership, and life skills, transferring them to the daily lives of the participants. At early ages, these improvements can be difficult to achieve, so the coach should create learning environments to improve these capabilities. TESPODEP is a well-structured intervention program that aims to overcome these limitations, providing useful guidelines for coaches. Some recommendations to coaches when intervening within the competitive sport could be as follows: (a) set two different types of goals, sportive goals, and life skill goals; (b) integrate PYD strategies into training tasks; (c) use the methodological strategies offered to facilitate fostering PYD and life skill learning; (d) make all players play all roles throughout the season and let them make their own decisions; and (e) keep a balance between sports results and PYD intervention.

This study has some limitations. The main limitation is the sample size, which, although it presents an adequate and sufficient size to obtain relevant information for the scientific community at a qualitative level, seems to be insufficient to obtain significant results at a quantitative level (Creswell and Creswell, 2018; Creswell and Plano Clark, 2018). It should also be noted that this study was carried out with only one experimental group, in a specific sociocultural and socioeconomic context, in a single sport, and in a single category (limited age range) and competitive level. In addition, all of the participants were girls. This means that it is not possible to determine whether there are differences between genders, ages, sports, levels of sports demand, or socioeconomic/sociocultural levels.

To alleviate the limitations of this study, the following lines for future research studies are recommended: (a) to increase the sample size to draw on statistical data that contribute to a better understanding of the research question; (b) to carry out the study in different team sports, ages, competitive levels, and/or socioeconomic/sociocultural levels to determine how TESPODEP works in different samples and settings; and (c) to include both genders in the sample to determine differences between genders in terms of life skill acquisition.

## Conclusions

Hybridizing TPSR and SE models made it possible to design a program adapted to the intervention context in which it was applied (competitive extracurricular sports) to foster PR and SR and teach volleyball technical/tactical aspects in grassroots sport. TESPODEP made adaption to the intervention context (extracurricular competitive youth mini-volleyball) possible, where the TPSR structure contributed to fostering PR and SR throughout the intervention, while SE helped to incorporate the key elements of team sports for transferring learning to competition and the daily lives of the participants. In general terms, the analysis and subsequent reflection on that analysis showed that the implementation of the hybrid program TESPODEP seemed to present positive effects in terms of the development of PR and SR in youth girl volleyball players in an extracurricular setting.

Regarding PR, both the perceptions of players and coach indicated that they developed skills related to autonomy, effort, and responsibility with respect to the equipment throughout the intervention program. Concerning SR, again, the perceptions of players and coach both indicated that they acquired skills related to respect and caring and helping others. The insights of both players and the coach seem to indicate that the group positively perceived their learning and development in the analyzed dimensions. Although the quantitative data do not agree with the findings of the interviews and field notes on the development of PR and SR, as there were no statistically significant differences between or within groups, the authors consider that the main reason for this lack of positive results may have been the limited sample, which was not sufficient to achieve significant differences, and thus, the qualitative results derived from the interviews and field notes are more reliable for drawing conclusions about this study.

This study can serve as a reference to promote the design and implementation of hybrid TPSR+SE intervention programs to foment PYD through competitive team sports. However, it is necessary to increase the research field about this type of program, incorporating successful strategies like MINDSCAPE (Whitley, 2021) to foster long-term behavioral changes and assess them through longitudinal studies (e.g., throughout a whole sports category). It is also essential to come to an understanding with the coach about the program design, in addition to providing prior training about its key elements, ensuring implementation fidelity. Furthermore, using triangulation is essential to enhance the validity and reliability of the data. In conclusion, the hybrid program TESPODEP seems to be effective to develop PR and SR in youth girl athletes in a competitive extracurricular youth sports context.

## Data Availability Statement

The raw data supporting the conclusions of this article will be made available by the authors, without undue reservation.

## Ethics Statement

The studies involving human participants were reviewed and approved by Pablo de Olavide University's Ph.D. Research Committee. Written informed consent to participate in this study was provided by the participants' legal guardian/next of kin.

## Author Contributions

AM-L, PC-B, and EH-H elaborated the conceptualization, the methodological framework, the data validation, and the original draft writing, including results, discussion, and conclusions. AM-L and EH-H carried out the data analysis. AG-d-A took charge of reviewing and editing the final manuscript. All authors have read and agreed to the published version of the manuscript.

## Conflict of Interest

The authors declare that the research was conducted in the absence of any commercial or financial relationships that could be construed as a potential conflict of interest.

## Publisher's Note

All claims expressed in this article are solely those of the authors and do not necessarily represent those of their affiliated organizations, or those of the publisher, the editors and the reviewers. Any product that may be evaluated in this article, or claim that may be made by its manufacturer, is not guaranteed or endorsed by the publisher.
