# Supplementary material for: Personal and Social Responsibility Development in a Volleyball Hybrid Positive Youth Development Program: A Mixed Methods Approach
Source: Front Psychol. 2021 Aug 11;12:675532. doi: 10.3389/fpsyg.2021.675532 (PMC8385124; doi:10.3389/fpsyg.2021.675532)
Supplement: Supplementary file 1 [file Table_1.DOCX]

Supplementary Material

# General characteristics of the TESPODEP program

Levels of responsibility and goals of each level

Level 1: Establishing a positive group climate

- Main goal: Establishing a positive climate among participants (safe physical and psychological atmosphere)
- Specific goals:
  - Collaborate in the establishment of a positive group climate through communication and trust between participants and coach.
  - Know and respect cohabitation rules and associated penalties.
  - Know and use tools to solve conflicts in a calm and autonomous way.

Level 2: Participation and effort

- Main goal: Fostering participation and effort in tasks and orientating motivation towards learning
- Specific goals:
  - Participate in all tasks, independent of the skill level.
  - Make an effort, persist and be attentive in tasks.
  - Show a high level of motivation and involvement in tasks during training.

Level 3: Self-direction

- Main goal: Developing self-direction and decision-making capabilities
- Specific goals:
  - Enhance self-confidence and self-perception.
  - Develop capabilities of planning, setting goals and making decisions.
  - Acquire strategies and resources to accomplish set goals.

Level 4: Helping others and leadership

- Main goal: Fostering social abilities involved in leadership roles
- Specific goals:
  - Reflect on the importance of interpersonal communication and use strategies to boost it when communicating to others.
  - Know the characteristics and differences between the communication styles and become aware of the importance of being assertive.
  - Become aware of own’s feelings and recognize others’ feelings.
  - Recognize the importance of cooperation to accomplish common goals and offering/requesting help.

Level 5: Transference

- Main goal: Promote transference of learned capabilities to real life
- Specific goals:
  - Apply learned capabilities in competitive situations within sport.
  - Apply learned capabilities outside the sportive context.

Strategies to integrate levels of responsibility within practice

- Level 1
  - Make the sides fair
  - Modify task rules to enhance participation of the whole group
  - Establish a unique team name
  - Create a unique battle cry and say it at the end of all training sessions and competitions
- Level 2
  - Self-modification of tasks
  - Self-paced challenges
  - Redefining success
  - Working with intensity scales
- Level 3
  - On-task independence
  - Setting personal goals
  - Create a personal plan to accomplish set goals
- Level 4
  - Use the different communication styles
  - Treasure hunt using elements of an effective interpersonal communication as keys
- Level 5
  - Reflect on the application of learned capabilities in different contexts of life

General methodological strategies

These strategies make the implementation of the program more consistent, independent of the level of responsibility.

- Setting expectations: The coach explains to the players what he/she expects of them.
- Giving opportunities for success: The session is structured in a way every player has the opportunity to success in tasks.
- Fostering social interaction: The tasks are structured in order to foster positive social interaction.
- Setting roles and tasks: The coach assigns specific roles and responsibilities to the players.
- Mastery-oriented learning: Training sessions must have a specific goal; instructions and feedbacks are oriented towards this goal.
- Leadership: The coach allows the players to lead and be in charge of the group.
- Giving voice: The coach gives voice to the players, letting them make decisions within training sessions.
- Role in the evaluation: The coach allows the players to have an active role in evaluation through group and self-reflections.
- Respect model: The coach is a model of behavior and respect.
- Transference: The coach talks to the players about the possibilities of transferring learned capabilities to other contexts.

Specific methodological strategies

These strategies have relation with one or several levels of responsibility, and their objective is to accomplish specific goals of the different levels.

- Group control routines: The coach proposes a daily routine that contributes to a better control of the group and increases the practice time.
- Guided practice: The coach leads the training session when a new element or content is introduced.
- Independent practice: The players with the coach and assistant coach roles are in charge of the training session.
- Conflict resolution: The coach teaches the players the steps for conflict resolution, to allow them to solve conflicts independently.
- Coach’s portfolio: The coach gives the player-coaches a portfolio with cards that explain the different technical-tactical elements and the steps for conflict resolution.
- Team identity: The coach fosters the creation of a unique group identity in order to generate feelings and memories that contribute to the development of a feeling of team membership in the players.

# Interview script

Previous Experience and other activities

1. Have you practiced volleyball before this year?
2. Do you do any other after-school activity apart from volleyball?

Perception about learning during the program

1. Do you think the training sessions have helped you to be able to lead other people within volleyball? And in your life outside of sports? Why?
2. Do you think the training sessions have made you learn to make better decisions when you have set a goal to achieve or when there has been a problem? Why?
3. Do you think the training sessions have helped you to be more responsible in trainings and competitions? And in your life outside of sports? Why?
4. Do you think the training sessions have helped you to be a better volleyball player? In which technical gesture do you think you have improved the most? And in which less? And in the matches?
5. Of the five levels that we have worked on, what do you think you have learned?
   1. Level 1: Establish a positive group climate
   2. Level 2: Participation and effort
   3. Level 3: Autonomy
   4. Level 4: Helping others and leadership
   5. Level 5: Transference
6. Do you think your parents have realized how much you have improved?
7. Do you think the coach has realized how much you have improved?
8. From what you have learned, what things have you used in your daily life?

Perception about the program

1. What do you think about the training sessions that the coach has done? And about the ones that you have done?
2. What do you think about trying to take care of the positive atmosphere of the group?
3. What do you think about trying to make you participate and make an effort in training sessions and matches?
4. What do you think about trying to make you autonomous and train on your own?
5. What do you think about trying to get you to cooperate with each other?
6. What do you think about trying to make you learn to communicate with others in an assertive way?
7. What do you think about the different roles you have had throughout the season? Do you think they have been carried out properly? Why?
   1. Coach.
   2. Assistant coach.
   3. Material manager.
   4. Cheerleader.
   5. Scorer.
8. What did you like the most about training sessions?
9. What did you like the least? How would you improve it?

Perception of the coach's work

1. What do you think of the coach's performance throughout the program?
2. Do you think the coach has had a good relationship with the team? Have you felt comfortable with him?
3. Do you think the coach has put in the effort and involved in the program?
4. What aspects did you like the most about the coach? Which ones would you change?

# Field notes’ model

| **Coach** | |  | | **Session nº** | | | |  | | **Date** | | |  |
| --- | --- | --- | --- | --- | --- | --- | --- | --- | --- | --- | --- | --- | --- |
| **Technical objective** | |  | | | | | | | | | | | |
| **Contents** | |  | | | | | | | | | | | |
| **Activities**  **(brief description)** | |  | | | | | | | | | | | |
|  | | | | | | | | | | | | | |
| **Responsibility level** | | 1 | 2 | | | | 3 | | 4 | | | 5 | |
| **Responsibility objective** | | A)  B) | | | | | | | | | | | |
| **Responsibility objective acquisition**  **(1-10)** | | A) X  B) X | | | | | | | | | | | |
| **SPECIFIC METHODOLOGICAL STRATEGIES** | | | | | | | | | | | | | |
| Group control routines | | | | | | Conflict resolution | | | | | | | |
| Guided practice | | | | | | Coach’s portfolio | | | | | | | |
| Independent practice | | | | | | Team identity | | | | | | | |
| **GENERAL METHODOLOGICAL STRATEGIES** | | | | | | | | | | | | | |
| Setting expectations | | | | | | Setting roles and tasks | | | | | | | |
| Giving opportunities for success | | | | | | Leadership | | | | | | | |
| Fostering social interaction | | | | | | Role in the evaluation | | | | | | | |
| Giving voice | | | | | | Respect model | | | | | | | |
| Mastery oriented learning | | | | | | Transference | | | | | | | |
| **METHODOLOGICAL FOUNDATIONS** | | | | | | | | | | | | | |
| Embedding | Transference | | | | Empowerment | | | | | | Being relational | | |
| OBSERVATIONS | | | | | | | | | | | | | |
| Session number, how does it go  Coach performance  Assistant coach performance  How they act together  Remaining roles performance  Group and self-reflection | | | | | | | | | | | | | |
